# Depression level and coping responses toward the movement control order and its impact on quality of life in the Malaysian community during the COVID-19 pandemic: a web-based cross-sectional study

**DOI:** 10.1186/s12991-021-00352-4

**Published:** 2021-05-24

**Authors:** Anne Yee, Nur ‘Aqilah Mohd Hodori, Yu-Zhen Tung, Po-Lin Ooi, Saiful Adni B. Abdul Latif, Husna Md Isa, Diana-Leh-Ching Ng, Chee-Shee Chai, Seng-Beng Tan

**Affiliations:** 1 Department of Psychological Medicine, Faculty of Medicine, University of Malaya, University Malaya Medical Centre, 50600 Kuala Lumpur, Malaysia; 2 Department of Medicine, Faculty of Medicine, University of Malaya, 50603 Kuala Lumpur, Malaysia; 3 Department of Clinical Oncology, Faculty of Medicine, University of Malaya, 50603 Kuala Lumpur, Malaysia; 4 Department of Palliative and Supportive Therapy, National Cancer Institute, 62250 Putrajaya, Malaysia; 5 Faculty of Cognitive Sciences and Human Development, University Malaysia Sarawak, 94300 Kota Samarahan, Sarawak Malaysia; 6 Department of Medicine, Faculty of Medicine and Health Science, University Malaysia Sarawak, 94300 Kota Samarahan, Sarawak Malaysia

**Keywords:** COVID-19 pandemic, Depression, Coping, Quality of life, Mental health, Coronavirus

## Abstract

**Background:**

Coronavirus 2019 disease (COVID-19) is a highly infectious disease prompting extreme containment measures, including lockdown, travel restrictions, social distancing, and stringent personal hygiene. This study investigates the depression level and coping responses toward the lockdown, referred as the movement control order (MCO) during COVID-19 pandemic in Malaysia and its impact on quality of life.

**Method:**

This cross-sectional study was conducted from April to May 2020. The outcomes were assessed using the Depression, Anxiety and Stress Scale–21, Coping Orientation to Problems Experienced Inventory, and World Health Organisation Quality of Life–BREF Scale (WHOQOL-BREF) in both English and validated Malay versions.

**Results:**

Mild-to-severe depression was found in 28.2% (*n* = 149) of the 528 respondents. Respondents with mild-to-severe depression were significantly younger (33.09 ± 10.08 versus 36.79 ± 12.47 years), without partner (71.8% versus 45.6%), lived in the red zone (85.9% versus 71.0%), and had lower household income as defined in the category of B40 (51.7% versus 39.3%) compared to those without depression (all *p* < 0.01). The avoidant coping score was significantly higher (25.43 ± 5.69 versus 20.78 ± 5.65), while the religious coping score was significantly lower (5.10 ± 2.07 versus 5.94 ± 2.11) among those with mild-to-severe depression compared to those without depression (both *p* < 0.001). Respondents with mild-to-severe depression also had significantly lower mean score in each domain of WHOQOL-BREF compare to those without depression [(physical health, 13.63 ± 2.66 versus 16.20 ± 2.11), (psychological, 12.5 ± 2.79 versus 16.10 ± 2.14), (social relationships, 12.17 ± 3.49 versus 15.28 ± 2.93), environment (14.50 ± 2.39 versus 16.21 ± 2.14), all *p* < 0.001] after controlling for age, marital status, zone, household income, and coping scores.

**Conclusion:**

COVID-19 lockdown had adverse mental health effects. Our study highlighted that approximately one in three individual experienced mild-to-severe depression during the nationwide MCO. The varied impact of the pandemic on mental health could be due to different population characteristics and coping strategies used. Identifying those at higher risk to develop depression during MCO for COVID-19 pandemic could help mental healthcare service providers to plan services for those susceptible, thereby mitigating the pandemic’s effect on quality of life.

## Background

The ongoing outbreak of Coronavirus 2019 disease (COVID-19) is the latest global health threat. It was first reported in humans in December 2019, in Wuhan, the capital city of Hubei province in China. COVID-19 is an infectious disease caused by the novel severe acute respiratory syndrome coronavirus 2 (SARS-CoV-2), an enveloped single-strand RNA virus of the *Coronaviridae* family [1]. COVID-19 is highly contagious and has spread rapidly worldwide, leading to a global health emergency of unprecedented scale. As of March 21, 2021, 118 million people have been infected with COVID-19 globally, with more than 2.7 deaths worldwide [2].

COVID-19 is highly transmissible through direct contact, droplets, and fomites [3]. The most effective method of containment is the practice of personal hygiene and curbing direct human contact [4]. The latter includes social distancing within communities and the unprecedented move to impose lockdown, movement control, and travel bans between cities, states, and countries.

Malaysia’s first COVID-19 case was reported in January 2020. Following a vast increment of COVID-19 cases, the nationwide lockdown, known as the movement control order (MCO), was enforced on March 18, 2020 with Malaysians instructed to stay at home, practice social distancing, and minimize contact with non-household members. Quarantine comprises the separation and restriction of movement of people potentially exposed to a contagious disease. The MCO differed from quarantine as it was widespread and involved people who might not have had contact with the virus. However, the impact of the MCO and quarantine might have had similar repercussions on psychological well-being.

Quarantine has been found to have a negative psychological impact, with studies showing that people quarantined during the epidemic of influenza, severe acute respiratory syndrome (SARS), middle east respiratory syndrome (MERS), and Ebola suffered from depression, anxiety, and post-traumatic stress disorder [5]. The duration of quarantine, fear of contagion, inadequate supplies, inadequate information, boredom, frustration, and sense of isolation were the stressors identified during the quarantine [5].

Different coping strategies during the pandemic may lead to different emotional impact. Coping strategies are methods involving thought or actions, which individuals adopt to manage stressors [6]. They are categorized according to their intended function, whether to resolve the stressful situation (problem-focused coping), reduce stress related to the stressful event (i.e., emotion-focused coping), or to avoid or approach the source of stress (approach versus avoidance-oriented coping). Reviews have reported that approach-oriented and emotion-focused coping strategies in stressful circumstances were associated with positive psychological and physical health outcomes. On the other hand, although avoidance coping strategies could be successful for short-term uncontrollable stressors, they have been linked to increased distress, particularly, if the stressors were chronic and uncontrollable [7]. Emotions are associated with specific coping strategies. Individuals who feel sad are more likely to use non-active coping methods such as avoiding or accepting the problem. In the beginning of the SARS outbreak, emotion-focused coping reduced sadness and anger in all age groups. The use of problem-focused coping reduced sadness in older adults [8]. A better understanding of the types of coping and associated factors could help moderate the impact of COVID-19 on depression among Malaysians.

A systemic review has reported mental health consequences during COVID-19 pandemic among COVID-19 patients, psychiatric patients, health care workers, and publics [9]. During the pandemic, COVID-19 patients seem to experience a high level of post-traumatic stress symptoms (PTSS) and depressive symptoms, while those with pre-existing psychiatric disorders report worsening of the psychiatric symptoms. Health care workers report increased depression, anxiety, psychological distress, and poor sleep quality; whereas public have lower psychological well-being and higher scores of anxiety and depression than before. In Italy, 3 weeks of COVID-19 lockdown have led to PTSS, depression, anxiety, insomnia, high perceived stress, and adjustment disorder among the general population [10]. However, as of the time of writing, no studies have examined the impact of COVID-19 or the implementation of the MCO on Malaysians’ mental health and quality of life. Therefore, this study aims to 1) determine the prevalence of depression during the MCO; 2) determine the associated factors and coping strategies between Malaysians with and without depression; and 3) evaluate the impact of the MCO on Malaysians’ quality of life.

## Methods

### Study design

This study was conducted between April and May 2020 during the first phase of the MCO. It was a nationwide cross-sectional study using respondent-driven sampling. Respondents were eligible if they were Malaysian citizens, aged 18 years old and above, able to read English or Malay, and resided in Malaysia during the MCO. Respondents with underlying chronic debilitating physical illness, psychiatric disorder, under investigation for COVID-19 or confirmed case of COVID-19 were excluded, because these characteristics could be confounding factors for depression per se.

### Sample size

The sample size was calculated based on a formula for cross-sectional study [sample size = *Z*_1-α_^2^ p[1−p]/d2], in which Z is confidence interval at 95%, *d* is margin of error of 5% and d is expected proportion of depression during COVID-19 pandemic at 19.8% based on a recent study [11]. Therefore, the minimum sample size for this study was 244 patients.

### Study procedures

We used a web-based cross-sectional survey to collect data to avoid the spread of SARS-CoV-2 by contact. Recruitment was conducted through advertisements posted on WhatsApp. Having read the instructions, the participants were asked to complete an online consent form to acknowledge that they had read and understood the study purpose, risks, and benefits. The anonymous self-administered online questionnaire took approximately 15–20 min to complete and was administered in both English and Malay. Respondents were not given any incentive to complete the survey.

### Ethics and consent

This anonymous online survey was conducted as per the Declaration of Helsinki and approved by the Medical Ethics Committee of the University Malaya Medical Centre (UMMC) (MREC ID NO202048-8477).

### Measures

The survey comprised a sociodemographic section and specific study instruments. The relevant sociodemographic data and COVID-19 related information were collected from the participants. The sociodemographic section included information on age, gender, religion, ethnicity, level of education, employment status, household income, and marital status. Household income was categorized according to the national income classification, with T20 as the top 20%, M40 as the middle 40%, and B40 as the lower 40% of family income in Malaysia [12].

The COVID-19-related information included respondents’ areas of residence according to the color-coding system assigned by the Ministry of Health. Areas were color-coded according to the number of cases: red zones having more than 41 cases, yellow zones having between 1 and 40 cases, and green zones having no cases [13].

It also included whether the respondents were frontline workers, defined as people, whose occupation required them to have direct contact with potential COVID-19 patients [14].

## Study instruments

### Depression, anxiety, and stress scale (DASS-21)

The DASS-21 is a 21-item questionnaire developed by Lovibond and Lovibond [15]. It is an abbreviated version of the original 42-item DASS-42 questionnaire developed by the same author. DASS-21 is a simple and concise self-administered tool that is used to screen for depression, anxiety, and stress. It has seven items per each domain of depression, anxiety, and stress. It has been validated in many languages, including Malay [16]. Higher total scores for each domain reflect the severity of the respective domains. The severity score for depression in DASS-21 is stratified into normal (0–9), mild (10–13), moderate (14–20), severe (21–27), and extremely severe (28 or more). The Cronbach’s alpha for depression subscale is 0.81. In this study, the respondents were defined as having depression if their DASS-21 depression score exceeded 9.

### Brief-COPE (Coping Orientation to Problems Experienced) Inventory

Brief-COPE is a 28-item self-rated questionnaire used to assess coping responses to stress [17]. The 14 subscales represent different patterns of coping, with each subscale comprising two items. Brief-COPE has Cronbach’s alpha of 0.70 for overall, and 0.44–0.89 for the 14 subscales. Both the English and Malay versions have been validated in Malaysia [18].

### World Health Organisation Quality of Life (WHOQOL)-BREF

This abbreviated tool was developed to measure the quality of life. The original tool, WHOQOL-100, although useful, was too lengthy to administer for practical use. The WHOQOL-BREF is a self-rated questionnaire containing 26 items that assess the quality of life in the four domains of physical health, psychological health, social relationships, and environment. Furthermore, two items measure overall quality of life and general health. The WHOQOL-BREF fits with the WHO definition of quality of life (QOL). Despite its brevity, it allowed researchers and clinicians to obtain information on the different domains of QOL. The WHOQOL-BREF domain scores correlate strongly with WHOQOL-100 domain scores. It also has good internal consistency, good discriminant validity, and test–retest reliability [19]. The overall Cronbach’s alpha was 0.89, with a range of 0.71–0.81 for each domain. The Malay version of the WHOQOL-BREF has been shown to have satisfactory psychometric properties [20].

### Statistical analysis

Analyses were conducted using SPSS version 23. The data were summarized using descriptive statistics. The non-normally distributed continuous variables in this study were transformed into normal distributed variables. The respondents were defined as having mild-to-severe depression if their DASS-21 depression score exceeded 9. Bivariate analyses were used to assess the association between sociodemographic data, COVID-19-related variables, and coping strategies (Brief-COPE) with depression. Specifically, Chi-squared test was used for categorical variables, Fisher’s exact test was used for categorical variables with 2 × 2 contingency table and one of the expected value being less than 5, and independent *t* test for continuous variables. The effect size was presented in phi for categorical variables, in which 0.2–0.29 = weak, 0.30–0.39 = moderate, 0.40–0.69 = strong, 0.70 or higher = very strong; and Cohen’s d for continuous variables, in which 0.20–0.46 = small, 0.50–0.79 = medium, and 0.80 or higher = large. Logistic regression method was used to analyze the significantly different demographic characteristics and coping strategies between patients with mild-to-severe depression and without depression. The relationship between each domain of WHOQOL-BREF as dependent variables and mild-to-severe depression as independent variable, while controlling the significant variables in logistic regression as covariates, was explored using Analysis of Covariance (ANCOVA). The statistical significance was set at *p* < 0.05 as determined using the two-tailed tests. Bonferroni correction, 0.05/number of hypothesis was also performed to counteract the problem of multi-testing.

## Results

According to the DASS-21 severity of depression level, 149 (28.2%) respondents were classified as mild-to-severe depressed. Of those, 55 (10.4%), 50 (9.5%), 23 (4.4%), and 21 (4.0%) were categorized as mild, moderate, severe, and extremely severe, respectively.

An independent-samples t test was conducted to compare the age, duration of MCO, household size, and Brief-COPE scale domain (approach, avoidant, religion, and humor) scores between respondents with mild-to-severe depression and without depression. Respondents with mild-to-severe depression were significantly younger than those without depression [33.09 ± 10.08 versus 36.79 ± 12.47 years, t (329.66) = 3.54, *p* < 0.001, Cohen’s *d* = 0.33]. There were also significant differences in the avoidant coping and religion scores for those with mild-to-severe depression compared to those without depression. For avoidant coping score, those with mild-to-severe depression had significantly higher score than those without depression [25.43 ± 5.69 versus 20.78 ± 5.65, t (526) =  − 8.501, *p* < 0.001, Cohen’s *d* = 0.82]. For religion coping score, those with mild-to-severe depression had significantly lower score than those without depression [5.10 ± 2.07 versus 5.94 ± 2.11, t (526) = 4.131, *p* < 0.001, Cohen’s *d* = 0.40]. No significant difference was found between the two groups for duration of MCO, household size, approach coping, and humor scores (Table 1).

**Table 1:** Demographic characteristics and coping response of respondents with and without mild-to-severe depression

	Without depression (*n* = 379)	With mild-to-severe depression (*n* = 149)	Df	*χ*^*2*^*, t*	*p *value
Age, years, mean ± SD	36.79 ± 12.47	33.09 ± 10.08	329.66	3.54	< 0.001
Duration of MCO, days, mean ± SD	48.67 ± 6.12	48.20 ± 6.08	526	0.79	0.430
Health quarantine,* n* (%)					
Yes	28 (7.4)	15 (10.1)	1	1.03	0.311
No	351 (92.6)	134 (89.9)			
Gender,* n* (%)					
Male	148 (39.1)	52 (34.9)	1	0.78	0.376
Female	231 (60.9)	97 (65.1)			
Ethnicity,* n* (%)					
Non-Malay	215 (56.7)	96 (64.4)	1	2.62	0.105
Malay	164 (43.3)	53 (35.6)			
Marital status,* n* (%)					
Without partner	173 (45.6)	107 (71.8)	1	29.40	< 0.001
With partner	206 (54.4)	42 (28.2)			
Religion,* n* (%)					
No religion	10 (2.6)	8 (5.4)	1	2.47	0.116
Have religion	369 (97.4)	140 (94.6)			
Zone,* n* (%)					
Green and yellow	110 (29.0)	21 (14.1)	1	12.78	< 0.001
Red	269 (71.0)	128 (85.9)			
Household income					
B40	149 (39.3)	77 (51.7)	1	6.68	0.010
M40 and T20	230 (60.7)	72 (48.3)			
Household size, mean ± SD	4.31 ± 2.13	4.13 ± 2.10	525	0.89	0.375
Employment status, *n* (%)					
Unemployed	104 (27.4)	31 (20.8)	1	2.47	0.116
Employed	275 (72.6)	118 (79.2)			
Frontliners,* n* (%)					
Yes	47 (12.4)	13 (8.7)	1	1.44	0.231
No	332 (87.6)	136 (91.3)			
Education level,* n* (%)					
Up to secondary	40 (10.6)	11 (7.4)	1	1.23	0.267
Tertiary	339 (89.4)	138 (92.6)			
Brief-COPE, mean ± SD					
Approach	33.12 ± 8.72	32.87 ± 6.07	385.78	0.37	0.710
Avoidant	20.78 ± 5.65	25.43 ± 5.70	526	− 8.50	< 0.001
Religion	5.94 ± 2.11	5.10 ± 2.07	526	4.13	< 0.001
Humor	4.45 ± 1.81	4.30 ± 0.1.67	291.37	0.89	0.377

*SD* standard deviation, *df* degree of freedom, ${X}^{2}$ Chi-square test*;* *t* *t *test

A Chi-square test of independence was calculated comparing the frequency of mild-to-severe depression disorder among respondents of different demographic characteristics. A significant interaction was found for respondents’ marital status, *X*^2^ (1, *N* = 528) = 29.40, *p* < 0.001, phi = − 0.24, zone, *X*^2^ (1, *N* = 528) = 12.78, *p* < 0.001, phi = 0.16, and household income, *X*^2^ (1, *N* = 528) = 6.68, *p* = 0.010, phi = − 0.11. Respondents with mild-to severe depression were more likely to be without partners (71.8%), living in the red zone (85.9%) and being in the B40 household income group (51.7%). No significant interaction was found for other demographic characteristics (Table 1).

The logistic regression model was statistically significant, χ^2^(6) = 135.10, *p* < 0.001. The model explained 32.5% (Nagelkerke R2) of the variance in mild-to-severe depression and correctly classified 78.60% of cases. Adopting avoidant coping (Odd ratio = 1.19, 95% CI = 1.14, 1.24, *p* < 0.001) and living in red-zone areas (Odd ratio = 3.21, 95% CI = 1.80, 5.73, *p* < 0.001) were significantly associated with more mild-to-severe depression among the respondents. Meanwhile, adopting religion coping (Odd ratio = 0.74, 95% CI = 0.66, 0.83, *p* < 0.001) and had partners (Odd ratio = 0.37, 95% CI = 0.21, 0.64, *p* < 0.001) were significantly associated with less mild-to-severe depression among the respondents. The respondents’ age and household income were not associated with mild-to-severe depression (Table 2).

**Table 2:** Logistic regression: factors associated with mild-to-severe depression

Variable	B	95% CI	SE	Odd ratio exp (B)	*p *value
Avoidant	0.17	1.14, 1.24	0.02	1.19	< 0.001
Religion	− 0.30	0.66, 0.83	0.06	0.74	< 0.001
Age	0.01	0.99, 1.03	0.01	1.00	0.450
Marital status	− 1.01	0.21, 0.64	0.28	0.37	< 0.001
Zone	1.17	1.80, 5.73	0.30	3.21	< 0.001
Income	− 0.08	0.58, 1.47	0.29	0.92	0.740

*SE* standard error, *CI* confidence interval

A one-way ANCOVA was conducted to determine the statistically significant difference between the WHOQOL-BREF domain (physical health, psychological, social relationships, and environment) scores on respondents with and without mild-to-severe depression, controlling for age, marital status, zone, household income, avoidant coping, and religion scores. WHOQOL-BREF domain scores had a significant effect on respondents with and without mild-to-severe depression after controlling for age, marital status, zone, household income, avoidant coping, and religion scores, of which those with mild-to-severe depression had lower score. For the physical health domain, F(1,519) = 74.60, adjusted mean difference = − 2.12, *p* < 0.001, partial *η*^2^ = 0.13; for the psychological domain, F(1,519) = 124.12, adjusted mean difference = − 2.71, *p* < 0.001, partial *η*^2^ = 0.19; for the social relationships domain, F(1,519) = 46.04, adjusted mean difference = − 2.26, *p* < 0.001, partial *η*^2^ = 0.08, and for the environment domain, F(1,519) = 24.63, adjusted mean difference =− 1.17, *p* < 0.001, partial *η*^2^ = 0.05. (Table 3).

**Table 3:** Comparison between respondents with and without mild-to-severe depression with regards to the WHOQOL-BREF domain scores

The WHOQOL-BREF domains, mean ± SD	With mild-to-severe depression	Without depression	Mean difference	Adjusted mean difference^a^ (95% CI)	*p* value
Physical health	13.63 ± 2.66	16.20 ± 2.11	− 2.57	− 2.12 (− 2.60, − 1.64)	< 0.001
Psychological	12.50 ± 2.79	16.10 ± 2.14	− 3.60	− 2.71 (− 3.19, − 2.23)	< 0.001
Social relationships	12.17 ± 3.49	15.28 ± 2.93	− 3.11	− 2.26 (− 2.91, − 1.61)	< 0.001
Environment	14.50 ± 2.39	16.21 ± 2.14	− 1.71	− 1.17 (− 1.64, − 0.71)	< 0.001

^a^Adjusted for age = 35.75, marital status = 0.47, zone = 0.75, household income = 0.57, avoidant = 22.08, and religion = 5.70

## Discussion

Depression is a major mental health problem that primarily disrupts mood and the ability to experience pleasure. This mood disruption is coupled with associated physical, emotional and cognitive symptoms, and behavioral changes. Our study showed that during the MCO for COVID-19 pandemic, approximately one-third of Malaysians were mild-to-severely depressed. Factors that increased Malaysians’ likelihood of being depressed were: being in younger age group, having no partner, living in the red zone during the MCO, having income categorized as B40, and having avoidant coping strategies. Religious coping and having a partner were associated with less depression during the MCO. Depression was associated with a significantly worse quality of life across all domains.

The proportion of respondents with mild-to-severe depression in our study was 28.2%, which was higher than the prevalence of depression during COVID-19 in other countries. Specifically, when compared with depression levels during the COVID-19 pandemic, a study in China reported the prevalence of depression in the general population to be 20.1% [11]. One possible explanation why our study’s prevalence of depression was higher was because it involved people who were in lockdown and affected by the MCO directly. In comparison, the study done by Huang and Zhao et al. was a general survey among the Chinese population that might not be directly affected by the lockdown in Hubei. In addition, our study was done some time after the MCO was implemented, and possibly, the concern and effect of the MCO towards normal daily life were more pronounced. If the survey was done during the initial phase of the MCO, the prevalence of depression might have been lower. Therefore, it will be useful for future studies conducted to assess the effect of the MCO over time on the depression level. The prevalence of depression in our study was more similar to the prevalence of depression during the SARS epidemic in Taiwan and Canada. Past studies analyzing the prevalence of depression during SARS in Taiwan showed a similar prevalence of depression (27.5%) [21]. A study conducted on the effects of quarantine during the SARS outbreak in Toronto also revealed a similar prevalence of depression (31.2%) [22]. The depression symptoms may persist even beyond the duration of this pandemic. Studies during the SARS outbreak found that quarantining was predictive of a high level of depressive episodes, even 3 years after the outbreak [23]. Although the prevalence of depression during this pandemic in Malaysia was similar to that in other countries, the prevalence of depression was higher than in the general population, which ranged from 6.7 to 14.4% [24]. These findings of higher mental health problems during the current pandemic are consistent with studies that have shown that public health emergencies could cause mental health problems [25, 26].

Our study found that being younger was associated with a higher risk of depressive symptoms. Other COVID-19 studies supported our findings. A nationwide survey in China during the COVID-19 crisis showed that young adults were more vulnerable to depression [27]. Previous studies conducted during the SARS outbreak also revealed younger people to be more at risk of depression [22, 23]. This might be explained by the young people’s increasing use of social media to obtain information. However, this behavior could be counterproductive, as such information could trigger stress. This was supported by a recent study that demonstrated that frequent social media exposure during the COVID-19 pandemic was associated with mental health problems [28]. Our study also showed that Malaysians who belonged to a lower economic class (B40) were more significantly associated with depression. This finding was consistent with studies conducted during the SARS outbreak, which showed that people with an annual household income of less than $40,000 had increased depressive symptoms [22]. Those from the lower socioeconomic class were more likely to work part-time, odd hourly rates, and did not have access to paid vacation and sick days [29]. Therefore, the restriction of movement due to MCO, resulting in the closure of many economic trades causing job insecurity, could harm psychological well-being [30, 31]. Meaningful social relationships are fundamental to a healthy human life [32]. The presence of healthy supportive relationships can have a positive impact on long-term health outcomes. Studies have shown that the emotional support provided by meaningful relationships enhances psychological well-being [33], while loneliness is a risk factor for depression [34]. Social isolation due to the MCO may make these effects more apparent. However, it is unknown whether loneliness was a cause of depressive symptoms in Malaysians. Therefore, future studies will be needed to assess the effect of the COVID-19 lockdown on loneliness. Our study showed that living in the red-zone area with many confirmed COVID-19 cases was associated with depression, and this finding was supported by another study conducted in China, which showed that living in areas with COVID-19 patients increases psychological stress such as anxiety and anger. The closer they were to confirmed cases, the more the risk of being infected, which caused more psychological distress [35]. Traditionally, disaster models have described a bull’s eye effect, which assumes that the psychological effects of a disaster are narrowed and geographically circumscribed near the area of disaster [36]. A study done during the SARS outbreak showed that the respondents in epidemic area were less anxious than those in non-epidemic area [37]. Recent studies have also shown that instead of only the geographical location, the appraisal of risk may be more helpful to explain observations on how disaster affects the psychological well-being [36]. It is likely that the geographical effect of the pandemic cannot be simply explained by one theory only, and future research will be needed to ascertain how the geographical distance to the epicentre of the COVID-19 pandemic predicts depression and other psychological distress [38].

Our study demonstrated that avoidant coping strategies predicted more depression, corroborating studies in adolescents that have shown the association between avoidant coping strategies with depression and anxiety [39]. Another study of pregnant minority women showed a correlation between avoidant coping and depression in pregnancy [40]. Although avoidant coping may reduce distress in the short-term, in the long run, approach coping strategies are associated with a more positive outcome if the stressor is chronic [7]. This is because avoidance coping may forestall more effective ways of coping or involve harmful behaviors such as substance abuse [7].

There are two types of religious coping, positive and negative. Positive religious coping means developing a positive relationship with God, and involve meditation, prayer, and reflecting on God’s to help in distressing times. Negative religious coping is, when one believes the affliction is a punishment from God, or blames God for the mishaps. The literature suggests that positive religious coping is related to positive psychological adjustment to stress, while negative religious coping was related to negative psychological adjustment to stress [41]. As our study found that religious coping reduced depression level, it is possible that Malaysians adopted positive religious coping during this pandemic. The slowing of the daily pace and having more time at home during the MCO may have enabled Malaysians to meditate, pray, or connect more with God. Previous research has shown that being more self-aware, having a sense of faith and empowerment, and living with meaning and hope improves well-being [42]. This positive religious coping strategy may help Malaysians to cope with the stress and uncertainties of the pandemic and, as a result, ameliorate symptoms of depression. In our study, the approach to coping strategies was not significantly associated with depressive symptoms. Strategies such as problem-solving, positive reappraisal, and active acceptance involve taking steps to ameliorate the negative effects of stressors. Even in isolation, individuals can partake in many activities that spark positive emotions, such as hobbies or learning a language. These behavioral activation activities are diverting and can spark positive emotions, which would hopefully build resilience [43]. Therefore, finding ways to engage with life during traumatic events can improve general psychological well-being [44].

Our study demonstrated that depression affects quality of life in all domains. This finding was consistent with a previous study, which showed that depressive symptoms were associated with impaired quality of life [45]. Patients with depressive symptoms had poorer functioning than patients with no depressive symptoms [45]. The psychosocial disability due to depressive symptoms was also seen to be affected by the severity of the symptoms. During more symptomatic periods, the disability was worse than during remission [46, 47]. The effect of depression on the quality of life during the pandemic was also shown by a study, which revealed that individuals who were quarantined or indirectly exposed to SARS experienced depressive symptoms due to the economic downturn and poor social support [48].

Our study has several strengths and limitations. It not only examined depression but also the coping strategies used by Malaysians during the MCO and the impact of the depression on the quality of life. Also, our study was ones among the few studies that looked at the effect of the MCO on the psychological health of the general population instead of focusing only on the psychological health of healthcare workers. The limitations were: first, due to the nature of the MCO implementation, coupled with the risk of contagion, our study was conducted using an online questionnaire. Thus, the non-IT literate population might have been excluded, especially those living in rural areas or with a poor educational background. In addition, the participants of this study were mostly young individuals, predominantly female and excluded individuals with chronic medical and psychiatric illness, which possibly could make the findings of this study biased. Furthermore, as the study was conducted in English and Malay, it excluded those not literate in these two languages. The non-random sampling method predisposed to a selection bias and might not represent the Malaysian population. Also, due to the cross-sectional method, no causality could be established between coping, depression, and quality of life. Some of the Brief-COPE dimensions needed to interpret with caution due to the low internal consistency. Our study found that religious coping lessened depression, but it was not certain whether it was positive or negative religious coping that protected individuals from depression. Therefore, future studies will be needed to attain a better picture of Malaysians’ ability to cope.

## Conclusion

The COVID-19 pandemic is a public health disaster that required an unprecedented method of containment, including the MCO. However, this method is not without its ramifications and our study revealed that the MCO for COVID-19 pandemic negatively affects depression among Malaysians. As the whole world continues to face the threat of COVID-19, with a risk of subsequent infection waves that may require re-implementation of the MCO, knowledge about its negative effects and risk factors for depressive symptoms will help national healthcare policymakers to implement strategies to mitigate this effect.

## Data Availability

The datasets used and/or analyzed during the current study are available with the corresponding author on reasonable request.

## Abbreviations

ANCOVA: Analysis of covariance
Brief-COPE: Coping Orientation to Problems Experienced Inventory
B40: Below 40% income earners
COVID-19: Coronavirus 2019 disease
DASS-21: Depression, Anxiety and Stress Scale–21
MCO: Movement control order
MERS: Middle-East respiratory syndrome
M40: Medium 40% income earners
SARS-CoV- 2: Severe acute respiratory syndrome coronavirus 2
SARS: Severe acute respiratory syndrome
T20: Top 20% income earners
WHO: World Health Organisation
WHOQOL-BREF: World Health Organisation Quality of Life–BREF Scale
QOL: Quality of life
